# Impact of farnesoid X receptor single nucleotide polymorphisms on hepatic decompensation and mortality in cirrhotic patients with portal hypertension

**DOI:** 10.1111/jgh.14700

**Published:** 2019-06-14

**Authors:** Georg Semmler, Benedikt Simbrunner, Bernhard Scheiner, Philipp Schwabl, Rafael Paternostro, Theresa Bucsics, Albert Friedrich Stättermayer, David Bauer, Matthias Pinter, Peter Ferenci, Michael Trauner, Mattias Mandorfer, Thomas Reiberger

**Affiliations:** ^1^ Division of Gastroenterology and Hepatology, Department of Internal Medicine III Medical University of Vienna Vienna Austria; ^2^ Vienna Hepatic Hemodynamic Lab Medical University of Vienna Vienna Austria; ^3^ Ludwig Boltzmann Institute for Rare and Undiagnosed Diseases Vienna Austria; ^4^ CeMM Research Center for Molecular Medicine of the Austrian Academy of Sciences Vienna Austria

**Keywords:** advanced chronic liver disease, cirrhosis, polymorphism, *rs35724*, *rs56163822*

## Abstract

**Background and Aim:**

The nuclear farnesoid X receptor (FXR) regulates critical pathways of hepatic metabolism, inflammation, and gut mucosal barrier. Thus, we investigated the association of FXR‐single nucleotide polymorphism (SNPs) with hepatic decompensation and liver‐related mortality in patients with advanced chronic liver disease.

**Methods:**

Two FXR‐SNPs (*rs56163822* G > T and *rs35724* G > C) were genotyped in a cohort of 402 prospectively characterized patients with hepatic venous pressure gradient (HVPG) ≥ 6 mmHg.

**Results:**

Only 19 patients (4.7%) harbored a *rs56163822* T‐allele and had less pronounced liver disease as indicated by lower Child–Pugh score (CPS, 6 ± 1 *vs* 7 ± 2 points, *P* = 0.034) and higher albumin levels (38.9 ± 4.9 *vs* 35.9 ± 5.9 g/L, *P* = 0.026). In contrast, *n* = 267 (66.4%) patients harbored minor *rs35724* allele (G/C or C/C) and had more advanced liver disease, as indicated by a higher model of end‐stage liver disease (11 ± 4 *vs* 10 ± 3, *P* = 0.016), while other baseline characteristics were similar across FXR‐SNP genotypes. In compensated CPS‐A patients, the *rs35724* minor allele was independently protective for the development of ascites (adjusted hazard ratio [aHR] = 0.411, 95% confidence interval (95% CI): 0.191–0.885; *P* = 0.023) and tended to reduce the risk of hepatic decompensation (aHR = 0.625, 95% CI: 0.374–1.044, *P* = 0.072) in multivariate analyses. Of note, transplant‐free survival was longer in patients with *rs35724* minor allele and HVPG ≥ 10 mmHg (at 5 years: 68.2% *vs* 55.8%, *P* = 0.047) and those with HVPG ≥ 16 mmHg (63.3% *vs* 44.0%, *P* = 0.021). After adjusting for established risk factors, the *rs35724* minor allele was independently associated with reduced liver‐related mortality in the overall cohort (aHR = 0.658, 95% CI: 0.434–0.998, *P* = 0.049), in compensated CPS‐A patients (aHR = 0.488, 95% CI: 0.252–0.946, *P* = 0.034), in patients with HVPG ≥ 10 mmHg (aHR = 0.547, 95% CI: 0.346–0.864, *P* = 0.010), and in patients with HVPG ≥ 16 mmHg (aHR = 0.519, 95% CI: 0.307–0.878, *P* = 0.014).

**Conclusion:**

The FXR‐SNP *rs35724* was associated with a reduced risk for development of ascites and liver‐related mortality in patients with advanced chronic liver disease.

## Introduction

The farnesoid X receptor (FXR) is a member of the nuclear receptor superfamily and main nuclear bile acid receptor that regulates the expression of key target genes in bile acid, lipid, and glucose metabolism.^1^, ^2^, ^3^ The receptor is highly expressed in the liver and ileum and is involved not only in metabolic pathways but also in hepatic inflammation and fibrosis.^4^, ^5^ Accordingly, FXR agonists have been shown to reduce liver fibrosis, vascular remodeling, and sinusoidal dysfunction, as well as portal hypertension in experimental studies.^6^, ^7^

Chronic liver disease progresses from fibrosis to cirrhosis, which leads to portal hypertension. Evidently, portal hypertension (hepatic venous pressure gradient [HVPG] ≥ 6 mmHg) is a major contributor to decompensating events, such as ascites, portal hypertensive bleeding, and hepatic encephalopathy (HE), which substantially increase the risk of mortality.^8^, ^9^, ^10^, ^11^

Importantly, liver disease progression shows substantial interindividual variability, and thus, research has focused on the identification of genetic factors accelerating the progression to cirrhosis and predisposing for the development of liver‐related events. Just recently, a genetic variant of the patatin‐like phospholipase domain containing 3 (PNPLA3) gene has been linked to increased risks of hepatic decompensation and mortality in patients who had already developed portal hypertension.^12^

The first link between genic variants of FXR and liver disease has been established by van Mill and coworkers,^13^ showing that the *rs61755050* (C > T) single nucleotide polymorphism (SNP) was associated with a higher frequency of intrahepatic cholestasis of pregnancy, while *rs56163822* (G > T) was not. Moreover, both variants were associated with reduced activity of the FXR pathway.^13^, ^14^

In other studies, *rs56163822* was associated with inflammatory bowel disease, a higher cholesterol reduction under rosuvastatin, and, most interestingly, with spontaneous bacterial peritonitis (SBP) in patients with cirrhosis and ascites.^15^, ^16^, ^17^

Little is known about the functional relevance of *rs35724* SNP (G > C). However, associations of the homozygous genotype (C/C) of this variant with cholelithiasis in male patients and of the heterozygous genotype (G/C) with body mass index were observed.^18^, ^19^

Despite the central role of FXR signaling in liver disease, information on the impact of FXR‐SNPs in (advanced) chronic liver disease is scarce. Therefore, we aimed to investigate the effect of FXR‐SNPs on (further) hepatic decompensation and mortality in patients with portal hypertension.

## Methods

### Patients

Four hundred two consecutive patients with portal hypertension (HVPG ≥ 6 mmHg) were tested for FXR‐SNPs (*rs56163822* G > T and *rs35724* G > C) between January 1, 2004, and June 30, 2014, were included in this retrospective analysis based on prospectively collected data. Patient characteristics and laboratory parameters at baseline and during clinical follow up were recorded.

### Definition of hepatic decompensation and transplant‐free survival

Patients entered the survival analyses at the time of HVPG measurement. Patients' medical records were reviewed for the following events that defined (further) hepatic decompensation at baseline and during follow up: large‐volume paracentesis, SBP, portal hypertensive bleeding, severe HE (West Haven grade 3/4), and liver‐related death. Any (further) hepatic decompensation was defined by one of these events occurring during follow up.

For calculation of liver‐related transplant‐free mortality and transplant‐free survival (TFS) time, patients were censored on the day of surgery if they underwent liver transplantation, on the day of non‐liver‐related death, and at the end of follow up.

### Determination of farnesoid X receptor single nucleotide polymorphisms

FXR *rs56163822* and *rs35724* genotyping was performed by a StepOnePlus Real‐Time PCR System (Applied Biosystems, Foster City, California, USA) using the TaqMan SNP Genotyping Assays C_25598386_10 and C_2366616_10 for *rs56163822* and *rs35724* (Thermo Fisher Scientific, Waltham, Massachusetts, USA).

### Hepatic venous pressure gradient measurements

The Vienna Hepatic Hemodynamic Laboratory at the Medical University of Vienna performed the HVPG measurements according to a standardized operating procedure.^20^, ^21^ HVPG measurements were performed in the absence of non‐selective beta‐blockers and nitrates. Clinically significant portal hypertension was defined by HVPG values ≥ 10 mmHg.^9^

### Statistics

Statistical analyses were performed using IBM spss Statistics 24 (spss Inc., Armonk, New York, USA) and GraphPad Prism 6 (GraphPad Software, La Jolla, California, USA). Continuous variables were reported as mean ± standard deviation or median and interquartile range (IQR), and categorical variables were shown as numbers (*n*) and proportions (%) of patients. Comparisons of continuous variables were performed using Student's *t*‐test or Mann–Whitney *U*‐test, as applicable. Group comparisons of categorical variables were performed using either Pearson's *χ* ^2^ or Fisher's exact test. The incidence of any (further) hepatic decompensation, large‐volume paracentesis, SBP, portal hypertensive bleeding, HE, and TFS were assessed using the Kaplan–Meier method and compared between FXR‐SNPs (*rs56163822* G/G *vs* G/T and *rs35724* G/G *vs* G/C and C/C) using the log–rank test and Gehan–Breslow–Wilcoxon test. Adjusted Cox regression analyses were used to determine independent prognostic factors for any (further) hepatic decompensation, large‐volume paracentesis, and transplant‐free liver‐related mortality. A two‐sided *P* value ≤ 0.05 was considered as statistically significant.

### Ethics

This study was approved by the ethics committee of the Medical University of Vienna (EK 1526/2017). All patients gave their written informed consent to genetic testing. The requirement of a written informed consent specific to this retrospective analysis was waived by the ethics committee.

## Results

### Baseline patient characteristics

In total, 402 patients with a mean age of 53.8 ± 11.2 years of predominant male gender (*n* = 305, 75.9%) were included. The main etiologies were viral hepatitis (*n* = 230, 57.2%), followed by (non‐)alcoholic fatty liver disease (*n* = 141, 35.1%), or other etiologies (*n* = 31, 7.7%). Three hundred thirteen patients (77.9%) had clinically significant portal hypertension (CSPH) with a mean HVPG of 16 ± 7 mmHg and mean model of end‐stage liver disease (MELD) of 11 ± 4 points. Two hundred twenty‐one patients (59.9%) had Child–Pugh score (CPS)‐A, 110 (29.8%) CPS‐B, and 38 (10.3%) CPS‐C, while in 33 patients, CPS could not be fully evaluated at baseline. Importantly, 298 patients (74.1%) showed compensated advanced chronic liver disease, and 104 patients (25.9%) had decompensated advanced chronic liver disease (dACLD) at baseline (Table 1).

**Table 1:** Baseline patient characteristics. *P* values <0.05 are written in bold

		*rs56163822*			*rs35724*		
	Overall cohort, *n* = 402	Wild type (G/G), *n* = 383 (95.3%)	Variant (G/T), *n* = 19 (4.7%)	*P* value	Wild type (G/G), *n* = 135 (33.6%)	Variants (G/C, C/C), *n* = 267 (66.4%)	*P* value
Age, years ±SD	53.8 ± 11.2	53.9 ± 11.2	53.6 ± 11.0	0.932	55.1 ± 11.2	53.2 ± 11.1	0.116
Sex, male/female (% male)	305/97 (75.9%)	291/92 (76.0%)	14/5 (73.7%)	0.819	98/37 (72.6%)	207/60 (77.5%)	0.275
Etiology				0.067			0.787
(N)AFLD (%)	141 (35.1%)	134 (35.0%)	7 (36.8%)		50 (37.0%)	91 (34.1%)
Viral (%)	230 (57.2%)	222 (58.0%)	8 (42.1%)		74 (54.8%)	156 (58.4%)
Others (%)	31 (7.7%)	27 (7.0%)	4 (21.1%)		11 (8.1%)	20 (7.5%)
Child–Pugh score,† points ± SD	7 ± 2	7 ± 2	6 ± 1	**0.034**	7 ± 2	7 ± 2	0.469
CPS‐A	221 (59.9%)	209 (59.2%)	12 (75.0%)	0.288	69 (58.0%)	152 (60.8%)	0.131
CPS‐B	110 (29.8%)	106 (30.0%)	4 (25.0%)		42 (35.3%)	68 (27.2%)
CPS‐C	38 (10.3%)	38 (10.8%)	0 (0%)		8 (6.7%)	30 (12.0%)
Decompensated liver disease	104 (25.9%)	99 (25.8%)	5 (26.3%)	1.000	34 (25.2%)	70 (26.2%)	0.823
MELD, points ± SD	11 ± 4	11 ± 4	10 ± 2	0.158	10 ± 3	11 ± 4	**0.016**
HVPG, mmHg ±SD	16 ± 7	16 ± 7	17 ± 5	0.378	16 ± 7	16 ± 7	0.978
CSPH, *n* (%)	313 (77.9%)	295 (77.0%)	18 (94.7%)	0.069	99 (73.3%)	214 (80.1%)	0.120
Albumin, g × L^−1^ ± SD	36.0 ± 5.9	35.9 ± 5.9	38.9 ± 4.9	**0.026**	36.5 ± 5.5	35.7 ± 6.0	0.200
Bilirubin, mg × dL^−1^ (IQR)	1.10 (0.74–1.87)	1.14 (0.73–1.89)	1.18 (0.93–1.53)	0.973	1.13 (0.70–1.63)	1.15 (0.75–2.03)	0.301
AST, U × L^−1^ (IQR)	65 (45–100)	65 (45–101)	56 (38–86)	0.415	62 (45–86)	70 (44–102)	0.180
ALT, U × L^−1^ (IQR)	52 (30–86)	51 (30–86)	56 (35–78)	0.774	51 (31–76)	53 (29–91)	0.803
GGT, U × L^−1^ (IQR)	123 (67–212)	123 (68–212)	89 (47–207)	0.472	118 (64–216)	128 (67–207)	0.564

*P* values <0.05 are written inbold.

† Information on Child–Pugh score is available in *n* = 369 (91.8%) patients.

(N)AFLD, (non)‐alcoholic fatty liver disease; ALT, alanine aminotransferase; AST, aspartate aminotransferase; CPS, Child–Pugh stage; CSPH, clinically significant portal hypertension; GGT, γ‐glutamyltransferase; HVPG, hepatic venous pressure gradient; IQR, interquartile range; MELD, model of end‐stage liver disease; SD, standard deviation.

### Clinical follow up including hepatic decompensation and mortality

During a median follow up of 885 (IQR: 502–1690) days, 228 patients (56.7%) experienced any (further) hepatic decompensation. Specifically, 88 patients (21.9%) required at least one large‐volume paracentesis, 72 (17.9%) were admitted to hospital due to severe HE, and 27 (6.7%) and 25 (6.2%) had one or more events of SBP and portal hypertensive bleeding during follow up, respectively. Additionally, 30 (7.5%) underwent liver transplantation, while 109 patients (27.1%) died (Table S1).

### Distribution of farnesoid X receptor single nucleotide polymorphisms and association with hepatic function

Overall, *rs56163822* wild type (G/G) was present in 383 patients (95.3%), while heterozygosity for the T‐allele (G/T) was detected in 19 patients (4.7%). None of the patients was homozygous for the T‐allele. When comparing patient characteristics at baseline between these two genotypes, CPS was significantly lower in patients harboring the T‐allele (6 ± 1 *vs* 7 ± 2 points, *P* = 0.034), while albumin levels were significantly higher (38.9 ± 4.9 *vs* 35.9 ± 5.9 g/L, *P* = 0.026). Interestingly, CPS‐A was overrepresented in carriers of the *rs56163822* T‐allele (75.0%), while only four (25.0%) patients had CPS‐B and no one CPS‐C. Of note, MELD score and HVPG levels were similar between C/C homozygotes and T‐allele carriers (Table 1).

Because patients without prior decompensation are of special interest when investigating the genetic influence on further disease progression, baseline characteristics of CPS‐A patients were analyzed separately with regard to FXR‐SNPs. This analysis revealed no differences in baseline characteristics between *rs56163822* genotypes (Table S2).

As for *rs35724*, prevalence of the homozygous wild type (G/G) was 33.6% (*n* = 135), with heterozygosity (G/C) and homozygosity (C/C) for the minor allele being observed in 49.3% (*n* = 198) and 17.2% (*n* = 69), respectively. Apart from a significantly higher MELD score (11 ± 4 *vs* 10 ± 3 points, *P* = 0.016) at baseline, no statistically significant differences were observed between *rs35724* G/G patients and patients with at least one minor allele (G/C and C/C).

A subgroup analysis of CPS‐A patients confirmed similar baseline characteristics in patients with *rs35724* G/G and at least one minor allele (G/C and C/C) (Table S2).

### Linkage between farnesoid X receptor single nucleotide polymorphisms and hepatic decompensation

**Figure 1:**
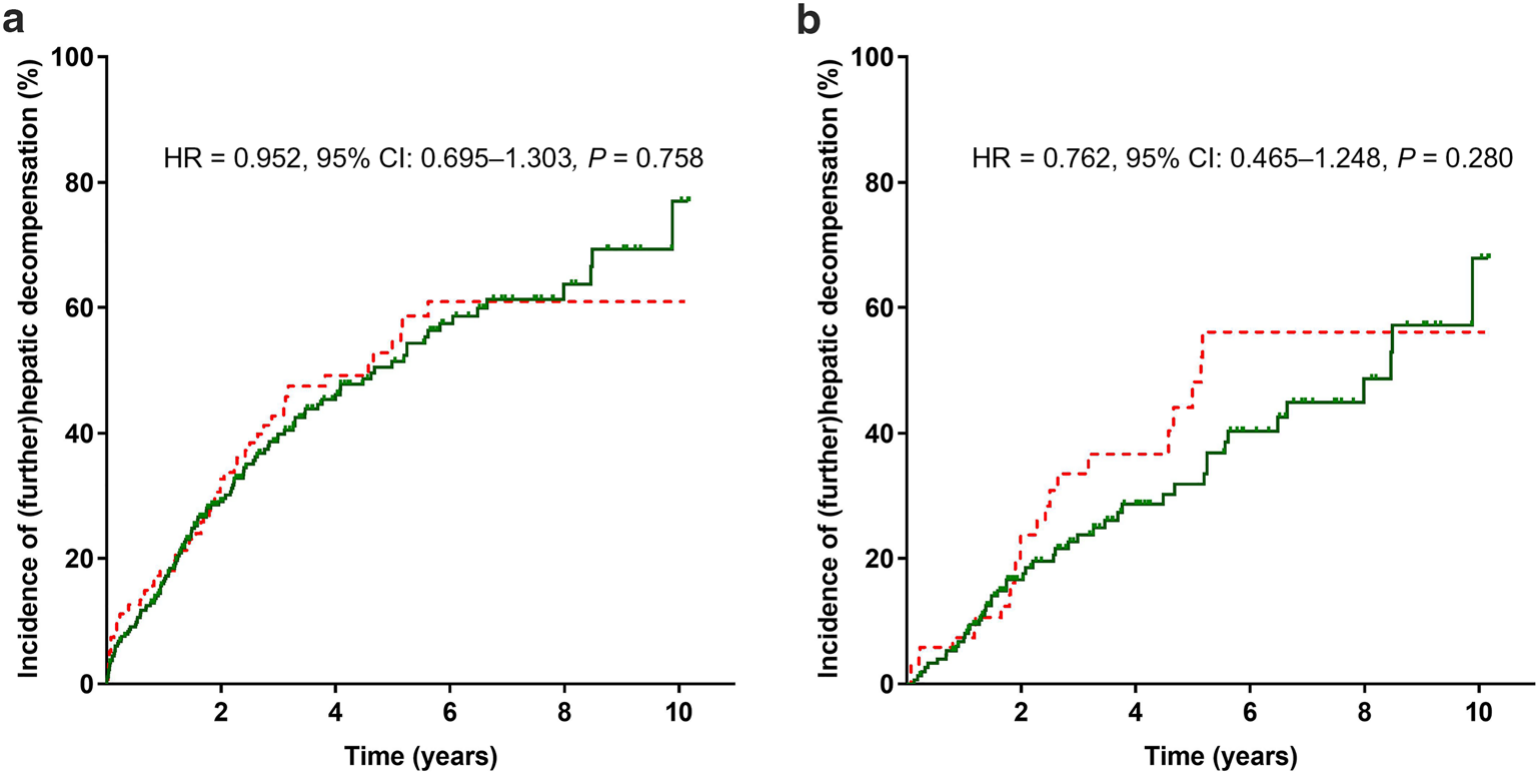
Kaplan–Meier analyses in patients with and without *rs35724* minor allele (G/C or C/C) on any (further) hepatic decompensation (a) in the overall cohort and (b) first decompensation in CPS‐A patients. 95% CI, 95% confidence interval; HR, hazard ratio. (a, b) 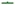, *rs35724* G/C or C/C; 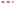, *rs35724* G/G. [Color figure can be viewed at http://wileyonlinelibrary.com]

The proportion of patients experiencing any (further) hepatic decompensation was comparable between patients with *rs56163822* G/G and G/T genotypes in the overall cohort (at 5 years: 53% *vs* 34%, hazard ratio [HR] = 0.671, 95% confidence interval [95% CI]: 0.297–1.516, *P* = 0.337) and in the subgroup of CPS‐A patients (at 5 years: 38% *vs* 26%, HR = 0.747, 95% CI: 0.234–2.385, *P* = 0.623; Fig. S1) (Fig. 1; Table 2).

**Table 2:** Cox regression analyses on the influence of *rs56163822* SNP (G/T), *rs35724* (G/C or C/C), and other parameters on any (further) hepatic decompensation (A) in the overall cohort and (B) on first decompensation in CPS‐A patients during follow up. *P* values <0.05 are written in bold

	(A) Overall cohort, *n* = 402			(B) CPS‐A patients, *n* = 221		
Risk factors for hepatic decompensation	aHR	95% CI	*P* value	aHR	95% CI	*P* value
Age, per 10 years	1.325	1.148–1.528	**< 0.001**	1.214	0.954–1.544	0.115
Male gender (*vs* female)	1.357	0.947–1.944	0.096	1.840	0.972–3.482	0.061
HVPG, per mmHg	1.047	1.024–1.070	**< 0.001**	1.070	1.021–1.123	**0.005**
MELD, per point	1.010	0.962–1.059	0.697	1.030	0.924–1.149	0.588
Albumin, per g/dL	0.929	0.903–0.955	**< 0.001**	0.938	0.880–0.999	**0.048**
*rs56163822* SNP (G/T *vs* wild type)	0.773	0.339–1.760	0.540	0.708	0.220–2.279	0.562
*rs35724* SNP (G/C or C/C *vs* wild type)	0.830	0.602–1.145	0.256	0.625	0.374–1.044	0.072

*P* values <0.05 are written inbold.

95% CI, 95% confidence interval; aHR, adjusted hazard ratio; CPS, Child–Pugh stage; HVPG, hepatic venous pressure gradient; MELD, model of end‐stage liver disease; SNP, single nucleotide polymorphism.

Similarly, patients carrying the *rs35724* minor allele had a comparable course of liver disease regarding any (further) hepatic decompensation in the overall cohort (at 5 years: 55% *vs* 51%, HR = 0.952, 95% CI: 0.695–1.303, *P* = 0.758) and the subgroup of CPS‐A patients (at 5 years: 49% *vs* 32%, HR = 0.762, 95% CI: 0.465–1.248, *P* = 0.280).

Moreover, no statistically significant differences were evident in analyses comparing the need for large‐volume paracentesis, SBP, portal hypertensive bleeding, or admission for severe HE during follow up in CPS‐A patients with or *rs35724* minor allele (Fig. S2).

Neither *rs56163822* T‐allele nor *rs35724* minor allele increased the risk for hepatic decompensation in the overall cohort in a Cox regression analysis adjusting for age, sex, HVPG, MELD, and albumin. Nevertheless, the presence of *rs35724* minor allele tended to reduce the risk of any first hepatic decompensation in patients with CPS‐A (adjusted hazard ratio [aHR] = 0.625, 95% CI: 0.374–1.044, *P* = 0.072) and was additionally identified as an independent protective factor for the requirement of large‐volume paracentesis during follow up (aHR = 0.411, 95% CI: 0.191–0.885, *P* = 0.023; Table S3).

### Impact of farnesoid X receptor single nucleotide polymorphisms on transplant‐free survival and liver‐related mortality

**Figure 2:**
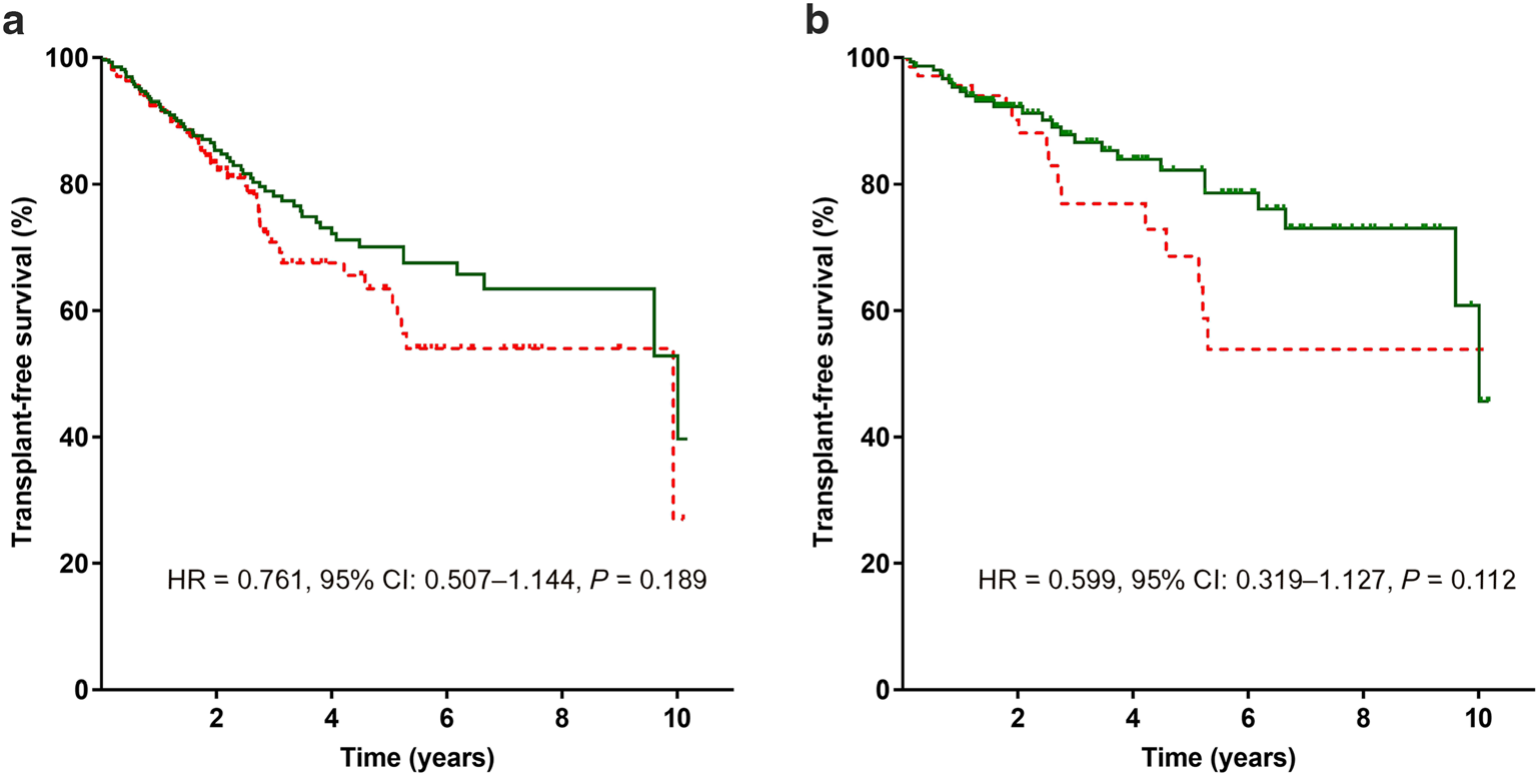
Transplant‐free survival* in patients with and without *rs35724* minor allele (G/C or C/C) (a) in the overall cohort and (b) in patients with CPS‐A. *Patients were censored at the day of liver transplantation, non‐liver‐related death, or end of follow up. 95% CI, 95% confidence interval; HR, hazard ratio. (a) 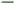, *rs35724* G/C or C/C; 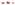, *rs35724* G/G. (b) 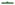, *rs35724* G/C or C/C; 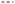, *rs35724* G/G. [Color figure can be viewed at http://wileyonlinelibrary.com]

To further investigate the influence of FXR‐SNPs on mortality in patients with portal hypertension, TFS was compared between genotypes. In line with previous analyses, no significant differences were observed between *rs56163822* genotypes neither in the overall cohort (at 5 years: 67% *vs* 80%, HR = 0.768, 95% CI: 0.280–2.104, *P* = 0.608) nor in patients with CPS‐A (at 5 years: 78% *vs* 86%, HR = 0.760, 95% CI: 0.180–3.205, *P* = 0.708; Fig. S3) (Table 3; Fig. 2).

**Table 3:** Cox regression analyses on the impact of *rs56163822* (G/T), *rs35724* (G/C or C/C), and other parameters on liver‐related mortality during follow up (A) in the overall cohort and (B) in CPS‐A patients. *P* values <0.05 are written in bold

	(A) Overall cohort, *n* = 402			(B) CPS‐A patients, *n* = 221		
Liver‐related mortality	aHR	95% CI	*P* value	aHR	95% CI	*P* value
Age, per 10 years	1.381	1.134–1.681	**0.001**	1.224	0.882–1.698	0.227
Male gender (*vs* female)	1.954	1.165–3.277	**0.011**	1.978	0.866–4.515	0.106
HVPG, per mmHg	1.020	0.985–1.056	0.257	1.067	0.999–1.140	0.054
MELD, per point	0.974	0.910–1.042	0.439	0.851	0.712–1.016	0.074
Albumin, per g/dL	0.904	0.871–0.940	**< 0.001**	0.932	0.853–1.018	0.119
*rs56163822* SNP (G/T *vs* wild type)	1.027	0.370–2.855	0.959	0.924	0.215–3.970	0.915
*rs35724* SNP (G/C or C/C *vs* wild type)	0.658	0.434–0.998	**0.049**	0.488	0.252–0.946	**0.034**

*P* values <0.05 are written inbold.

95% CI, 95% confidence interval; aHR, adjusted hazard ratio; CPS, Child–Pugh stage; HVPG, hepatic venous pressure gradient; MELD, model of end‐stage liver disease; SNP, single nucleotide polymorphism.

For *rs35724* SNP, TFS did not differ between homozygous wild type (G/G) patients and carriers of the C‐allele (at 5 years: 63% *vs* 70%, HR = 0.761, 95% CI: 0.507–1.144, *P* = 0.189). However, in CPS‐A patients, the presence of the minor allele was associated with a trend towards a longer TFS (at 5 years: 69% *vs* 82%, HR = 0.599, 95% CI: 0.319–1.127, *P* = 0.112).

Moreover, Cox regression analysis adjusted for age, sex, HVPG, MELD, and albumin revealed an independent association between presence of *rs35724* minor allele and reduced liver‐related mortality both in the overall cohort (aHR = 0.658, 95% CI: 0.434–0.998, *P* = 0.049) and in CPS‐A patients (aHR = 0.488, 95% CI: 0.252–0.946, *P* = 0.034) with *rs35724* minor allele being the only factor significantly associated with liver‐related mortality in CPS‐A patients.

Besides, Kaplan–Meier analysis indicated tendencies towards longer TFS in female patients (at 5 years: 56% *vs* 83%, HR = 0.376, 95% CI: 0.148–0.959, *P* = 0.041) and female patients with CPS‐A (at 5 years: 58% *vs* 88%, HR = 0.289, 95% CI: 0.068–1.234, *P* = 0.094; Fig. S4).

### Subgroup analysis of patients stratified by hepatic venous pressure gradient

**Figure 3:**
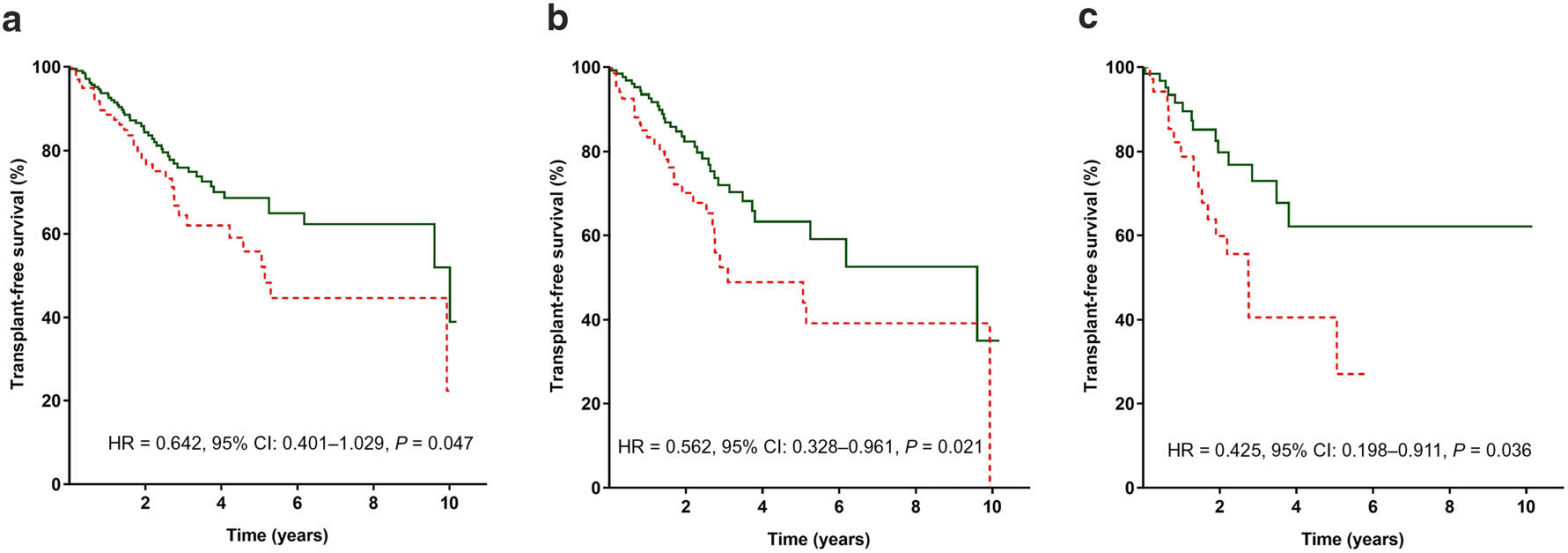
Transplant‐free survival* in patients with and without *rs35724* minor allele (G/C or C/C) (a) in patients with clinically significant portal hypertension (CSPH; HVPG ≥ 10 mmHg), (b) in patients with HVPG ≥ 16 mmHg, and (c) in patients with high‐risk CSPH (HVPG > 20 mmHg). *Patients were censored at the day of liver transplantation, non‐liver‐related death, or end of follow up. 95% CI, 95% confidence interval; CSPH, clinically significant portal hypertension; HR, hazard ratio; HVPG, hepatic venous pressure gradient. (a–c) 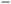, *rs35724* G/C or C/C; 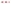, *rs35724* G/G. [Color figure can be viewed at http://wileyonlinelibrary.com]

When analyzing patients separately according to their HVPG, the distribution of *rs35724* minor alleles (G/C or C/C) was comparable between the HVPG strata: mild portal hypertension (HVPG 6–9 mmHg), HVPG 10–20 mmHg, and HVPG > 20 mmHg, *P* = 0.175; Table S4). While TFS did not differ between genotypes in patients with HVPG 6–9 mmHg and 10–20 mmHg, a potential survival benefit was evident in patients with CSPH (HVPG ≥ 10 mmHg; at 5 years: 68.2% *vs* 55.8%, HR = 0.642, 95% CI: 0.401–1.029, *P* = 0.047), patients with HVPG ≥ 16 mmHg (at 5 years: 63.3% *vs* 44.0%, HR = 0.562, 95% CI: 0.328–0.961, *P* = 0.021), and patients with high‐risk CSPH (HVPG > 20 mmHg, at 5 years: 62.1% *vs* 40.5%, HR = 0.425, 95% CI: 0.198–0.911, *P* = 0.036) (Table 4, Fig. 3).

**Table 4:** Cox regression analyses on the influence of *rs56163822* (G/T), *rs35724* (G/C or C/C) genotypes, and other parameters on liver‐related transplant‐free mortality during follow up (A) in patients with clinically significant portal hypertension (CSPH; HVPG ≥ 10 mmHg) and (B) in patients with HVPG ≥ 16 mmHg. *P* values <0.05 are written in bold

	(A) CSPH, *n* = 313			(B) HVPG ≥ 16 mmHg, *n* = 198		
Liver‐related mortality	aHR	95% CI	*P* value	aHR	95% CI	*P* value
Age, per 10 years	1.379	1.113–1.709	**0.003**	1.355	1.050–1.748	**0.019**
Male gender (*vs* female)	2.463	1.374–4.414	**0.002**	2.240	1.144–4.383	**0.019**
HVPG, per mmHg	1.017	0.974–1.062	0.443	1.003	0.940–1.070	0.939
MELD, per point	0.963	0.895–1.037	0.320	.917	0.837–1.005	0.064
Albumin, per g/dL	0.900	0.865–0.937	**< 0.001**	0.902	0.855–0.951	**< 0.001**
*rs56163822* SNP (G/T *vs* wild type)	1.044	0.374–2.915	0.935	1.500	0.514–4.378	0.459
*rs35724* SNP (G/C or C/C *vs* wild type)	0.547	0.346–0.864	**0.010**	0.519	0.307–0.878	**0.014**

*P* values <0.05 are written inbold.

95% CI, 95% confidence interval; aHR, adjusted hazard ratio; CSPH, clinically significant portal hypertension; HVPG, hepatic venous pressure gradient; HVPG, hepatic venous pressure gradient; MELD, model of end‐stage liver disease; SNP, single nucleotide polymorphism.

However, Cox regression analysis adjusted for age, sex, HVPG, MELD, and albumin revealed an independent association between presence of *rs35724* minor allele and reduced liver‐related transplant‐free mortality in patient with CSPH (HR = 0.425, 95% CI: 0.198–0.911), *P* = 0.036) and in patients with HVPG ≥ 16 mmHg (aHR = 0.519, 95% CI: 0.307–0.878, *P* = 0.014).

The risk of (further) hepatic decompensation did not differ between genotypes in all HVPG subcategories (Fig. S5). The hemodynamic response rate to non‐selective beta‐blocker therapy was comparable between *rs35724* variants (58.5% of patients with G/G and 59.3% with G/C or C/C, *P* = 0.935). Noteworthy, the relative change of HVPG was also similar with regard to the *rs35724* SNPs, G/G: median −17.0% (IQR: −23.8–[−3.0%]) *versus* minor alleles: −15.8% (IQR: −31.8–[−3.4%]), *P* = 0.976.

## Discussion

For a better understanding of modulators of liver disease progression, genetic factors have been increasingly investigated: For example, the PNPLA3 *rs738409* (C > G) SNP has been associated with an increased risk for hepatic decompensation and liver‐related mortality.^12^, ^22^ Additionally, the G‐allele is associated with steatosis, liver fibrosis, and disease activity in non‐alcoholic fatty liver disease^23^ and viral hepatitis.^24^, ^25^

In the present study, we aimed to explore the potential impact of two FXR‐SNPs on hepatic decompensation and mortality in patients with portal hypertension. Interestingly, the presence of *rs35724* minor allele was independently associated with reduced risk for liver‐related mortality in the overall cohort, in the subgroup of CPS‐A patients, and in patients with CSPH, severe portal hypertension (HVPG ≥ 16 mmHg), and high‐risk portal hypertension (HVPG > 20 mmHg). Finally, patients harboring the *rs35724* minor allele had a reduced requirement for large‐volume paracentesis.

The FXR‐SNP *rs56163822* G/T is prevalent in 2.5% of European population and 12.1% of Chinese patients and associated with reduced activity of FXR, but here, we did not find a significant clinical impact of this SNP in our cohort with a prevalence of 4.7% of the minor allele.^13^, ^14^ However, because FXR agonists have demonstrated anti‐fibrotic effects and a reduction of portal pressure in animal models, one may hypothesize that genotypes leading to a downregulation of FXR (such as *rs56163822* G/T) increase the risks of hepatic decompensation and mortality.^6^, ^7^, ^13^ Although we could confirm the prevalence of 4.7% in a Caucasian population, the low number of patients with heterozygous genotype substantially limits the conclusions that can be drawn from this study. However, a significantly lower CPS hints towards a protective effect on disease progression. This is in contrast to the cross‐sectional study of Lutz *et al*. reporting a higher prevalence of *rs56163822* G/T in patients with SBP compared with those without (including a total of 11 patients with this genotype).^17^ Although we were able to include a higher number of thoroughly characterized patients in our longitudinal study, a definite statement regarding the impact of this SNP on SBP incidence cannot be made based on our cohort.

The *rs35724* G/G, G/C, and C/C genotypes were reported to be prevalent in 37%/45%/17% in European patients, which was confirmed by our study.^15^ While the functional effect of the FXR *rs35724* SNP is currently unknown, one study reported a higher prevalence of the C/C genotype in male gallstone carriers, although there was no impact on the expression of genes involved in bile synthesis and transport.^18^ Our study uncovered a significant association of *rs35724* minor allele and improved survival in the overall cohort and subgroups of different disease severity. These findings highlight the potential “hepatoprotective” effect of the *rs35724* SNP on disease progression leading to a less pronounced course of liver disease as compared with patients with the homozygous wild type. In line with our observations, Mueller *et al*. (2017) reported a better liver function and improved survival in 341 patients with dACLD and ascites harboring *rs35724* minor allele.^26^ Just recently, Grimaudo *et al*. reported significantly higher hepatic FXR mRNA levels associated with the *rs35724* minor alleles—together with higher levels of circulating cholesterol and lower carotid artery intima‐media thickness of the common carotid arteries in a cohort of 124 patients biopsy‐proven non‐alcoholic steatohepatitis (NASH). Moreover, this variant was protective against severity of steatohepatitis and fibrosis.^27^

These reports support our hypothesis of a protective role of *rs35724* minor allele on liver disease progression and suggest an upregulation/activation of FXR signaling by this genotype, thus being a “gain‐of‐function” mutation.

Of note, the influence of the genetic background may be easier to detect in CPS‐A patients than in the overall cohort because these patients represent a rather homogenous cohort of patients without prior decompensation, in contrast to patients with dACLD, in whom non‐genetic, extrahepatic factors may have a more profound impact on the course of liver disease.^28^, ^29^, ^30^ Accordingly, less consistent results in the overall cohort seem reasonable. Despite the association between longer TFS and *rs35724*, minor allele was more pronounced in female patients. The results of our study regarding female subgroup must be interpreted with caution due to the limited sample size. Because no gender‐specific studies evaluating different expression of FXR in male and female have been carried out so far, further gender‐specific studies are warranted to investigate the specific functional consequences of this variant in female *versus* male patients.

Some limitations need to be acknowledged when interpreting the results of this study: Despite the retrospective design, patients were prospectively characterized at the timepoint of HVPG measurement, and clinical events during follow up were carefully assessed during scheduled clinical visits. Second, the statistical power to detect a clinically meaningful impact of *rs56163822* genotype was limited, because the number of patients harboring the *rs56163822* G/T genotype was small. Similarly, the results of subgroup analyses need to be interpreted with caution due to limited sample size in some analyses.

In conclusion, we are the first to investigate the impact of two FXR‐SNP (*rs56163822* and *rs35724*) on hepatic decompensation and liver‐related mortality in patients with portal hypertension. While we found prognostic value of the *rs35724* genotype for hepatic decompensation and mortality, further studies are needed to explore the underlying mechanisms and to confirm these results. However, the influence of this variant should be taken into account in future studies evaluating the effect of FXR agonists in humans.

## Supporting information


**Table S1.** Comparison of relative incidence of hepatic decompensation between FXR‐SNPs.
**Table S2.** Baseline characteristics and follow‐up in CPS‐A patients.
**Table S3.** Cox regression analysis on the influence of *rs56163822* SNP (G/T) and *rs35724* SNP (G/C or C/C), as well as other factors for the requirement of large‐volume paracentesis in CPS‐A patients.
**Table S4.** Distribution of *rs35724* variants among patients stratified by HVPG levels.
**Figure S1.** Kaplan‐Meier analyses on any (further) hepatic decompensation A in patients with and without *rs56163822* SNP in the overall cohort and B in patients with CPS‐A.
**Figure S2.** Kaplan‐Meier analyses on the incidence of A large‐volume paracentesis, B hepatic encephalopathy, C spontaneous bacterial peritonitis and D portal hypertensive bleeding in patients with CPS‐A and *rs35724* minor allele.
**Figure S3.** Transplant‐free survival* in patients with and without *rs56163822* SNP A in the overall cohort and B in patients with CPS‐A.
**Figure S4.** Kaplan Meier analyses on transplant‐free survival A in female patients and B in female CPS‐A patients with *rs35724* SNP minor allele.
**Figure S5.** Kaplan‐Meier analyses on (further) hepatic decompensation A in patients with mild portal hypertension (HVPG 6‐9mmHg), B in patients with HVPG 10‐20mmHg, C in patients with high risk CSPH (HVPG >20mmHg) as well as D in patients with clinically significant portal hypertension (CPSH, HVPG ≥10mmHg) and E HVPG ≥16mmHg, stratified according to presence of *rs35724* minor allele.
