# Supplementary material for: Impact of farnesoid X receptor single nucleotide polymorphisms on hepatic decompensation and mortality in cirrhotic patients with portal hypertension
Source: J Gastroenterol Hepatol. 2019 Jun 14;34(12):2164–72. doi: 10.1111/jgh.14700 (PMC6973125; doi:10.1111/jgh.14700)
Supplement: Supplementary file 1 — Table S1. Comparison of relative incidence of hepatic decompensation between FXR‐SNPs. Table S2. Baseline characteristics and follow‐up in CPS‐A patients. Table S3. Cox regression analysis on the influence of rs56163822 SNP (G/T) and rs35724 SNP (G/C or C/C), as well as other factors for the requirement of large‐volume paracentesis in CPS‐A patients. Table S4. Distribution of rs35724 variants among patients stratified by HVPG levels. Figure S1. Kaplan‐Meier analyses on any (further) hepatic decompensation A in patients with and without rs56163822 SNP in the overall cohort and B in patients with CPS‐A. Figure S2. Kaplan‐Meier analyses on the incidence of A large‐volume paracentesis, B hepatic encephalopathy, C spontaneous bacterial peritonitis and D portal hypertensive bleeding in patients with CPS‐A and rs35724 minor allele. Figure S3. Transplant‐free survival* in patients with and without rs56163822 SNP A in the overall cohort and B in patients with CPS‐A. Figure S4. Kaplan Meier analyses on transplant‐free survival A in female patients and B in female CPS‐A patients with rs35724 SNP minor allele. Figure S5. Kaplan‐Meier analyses on (further) hepatic decompensation A in patients with mild portal hypertension (HVPG 6‐9mmHg), B in patients with HVPG 10‐20mmHg, C in patients with high risk CSPH (HVPG >20mmHg) as well as D in patients with clinically significant portal hypertension (CPSH, HVPG ≥10mmHg) and E HVPG ≥16mmHg, stratified according to presence of rs35724 minor allele. [file JGH-34-2164-s001.docx]

**SUPPLEMENT**

**Supplementary Table-1.** Comparison of relative incidence of hepatic decompensation between FXR-SNPs.

|  |  | ***rs56163822*** | | ***rs35724*** | |
| --- | --- | --- | --- | --- | --- |
|  | **Overall cohort, n=402** | **Wildtype (G/G), n=383** | **SNP (G/T), n=19** | **Wildtype (G/G), n=135** | **SNP (G/C, C/C), n=267** |
| Median duration of follow-up (months) | 29 (16-56) | 29 (17-55) | 30 (13-63) | 27 (17-56) | 29 (16-55) |
| Large volume paracentesis during FU (%) | 88 (21.9%) | 83 (21.7%) | 5 (26.3%) | 29 (21.5%) | 59 (22.1%) |
| Severe HE during FU (%) | 72 (17.9%) | 69 (18.0%) | 3 (15.8%) | 27 (20.0%) | 45 (16.9%) |
| SBP during FU (%) | 27 (6.7%) | 25 (6.5%) | 2 (10.5%) | 5 (3.7%) | 22 (8.2%) |
| Hypertensive bleeding during FU (%) | 25 (6.2%) | 23 (6.0%) | 2 (10.5%) | 8 (5.9%) | 17 (6.4%) |
| Any (further) hepatic decompensation during FU (%) | 228 (56.7%) | 219 (57.2%) | 9 (47.4%) | 73 (54.1%) | 155 (58.1%) |
| Liver transplantation during FU (%) | 30 (7.5%) | 28 (7.3%) | 2 (10.5%) | 7 (5.2%) | 23 (8.6%) |
| Liver-related death during FU (%) | 109 (27.1%) | 105 (27.4%) | 4 (21.1%) | 39 (28.9%) | 70 (26.2%) |

Abbreviations: IQR – interquartile range; FU – follow up; HE – hepatic encephalopathy; SBP – spontaneous bacterial peritonitis

**Supplementary Table-2.** Baseline characteristics and follow-up in CPS-A patients

|  |  | ***rs56163822*** | | | ***rs35724*** | | |
| --- | --- | --- | --- | --- | --- | --- | --- |
|  | **CPS-A, n=221** | **Wildtype (G/G), n=209** | **Polymorphism (G/T), n=12** | *P value* | **Wildtype (G/G), n=69** | **Polymorphism (G/C, C/C), n=152** | *P value* |
| Age, years ±SD | 53.4±11.0 | 53.7±11.1 | 49.8±9.8 | *0.237* | 54.2±10.7 | 53.1±11.2 | *0.503* |
| Sex, male/female (% male) | 168 (76.0%) | 161 (77.0%) | 7 (58.3%) | *0.165* | 49 (71.0%) | 119 (78.3%) | *0.240* |
| Etiology |  |  |  | *0.057* |  |  | *0.357* |
| - (N)AFLD (%) | 49 (22.2%) | 46 (22.0%) | 3 (25.0%) |  | 18 (26.1%) | 31 (20.4) |  |
| - Viral (%) | 155 (70.1%) | 149 (71.3%) | 6 (50.0%) |  | 44 (63.8%) | 111 (73.0%) |  |
| - Others (%) | 17 (7.7%) | 14 (6.7%) | 3 (25.0%) |  | 7 (10.1%) | 10 (6.6%) |  |
| MELD, points ±SD | 9±2 | 9±2 | 9±2 | *0.515* | 8±2 | 9±3 | *0.205* |
| HVPG, mmHg ±SD | 13±5 | 13±6 | 15±3 | *0.054* | 13±6 | 13±5 | *0.358* |
| CSPH, n (%) | 148 (67.0%) | 137 (65.6%) | 11 (91.7%) | *0.110* | 41 (59.4%) | 107 (70.4%) | *0.108* |
| Albumin, g x L^-1^ ±SD | 38.9±4.0 | 38.8±4.0 | 39.7±4.2 | *0.456* | 39.2±4.0 | 38.7±4.0 | *0.458* |
| Bilirubin, mg x dL^-1^ (IQR) | 0.93 (0.64-1.25) | 0.90 (0.63-1.25) | 1.10 (0.88-1.56) | *0.180* | 1.02 (0.64-1.28) | 0.89 (0.63-1.25) | *0.276* |
| Large volume paracentesis during FU (%) | 32 (14.5%) | 29 (13.9%) | 3 (25.0%) | *0.388* | 12 (17.4%) | 20 (13.2%) | *0.407* |
| Severe HE during FU (%) | 22 (10.0%) | 20 (9.6%) | 2 (16.7%) | *0.340* | 9 (13.0%) | 13 (8.6%) | *0.301* |
| SBP during FU (%) | 5 (2.3%) | 4 (1.9%) | 1 (8.3%) | *0.246* | 2 (2.9%) | 3 (2.0%) | *0.649* |
| Hypertensive bleeding during FU (%) | 12 (5.4%) | 10 (4.8%) | 2 (16.7%) | *0.132* | 4 (5.8%) | 8 (5.3%) | *1.000* |
| Any first hepatic decompensation during FU (%) | 71 (32.1%) | 68 (32.5%) | 3 (25.0%) | *0.756* | 24 (34.8%) | 47 (30.9%) | *0.569* |
| (Liver related)-death during FU (%) | 45 (20.4%) | 43 (20.6%) | 2 (16.7%) | *1.000* | 16 (23.2%) | 29 (19.1%) | *0.482* |

Abbreviations: CPS – Child Pugh stage; SD – standard deviation; (N)AFLD – (non)-alcoholic fatty liver disease; MELD – model of end stage liver disease; HVPG – hepatic venous pressure gradient; CSPH – clinically significant portal hypertension; IQR – interquartile range; FU – follow up

**Supplementary Table-3.** Cox regression analysis on the influence of *rs56163822* SNP (G/T) and *rs35724* SNP (G/C or C/C), as well as other factors for the requirement of large-volume paracentesis in CPS-A patients.

| **Patient characteristics** | **Large volume paracentesis** | | |
| --- | --- | --- | --- |
|  | **aHR** | **95%CI** | ***P value*** |
| **Age, per 10 years** | 1.291 | 0.890-1.872 | 0.178 |
| **Male gender (vs. female)** | 3.588 | 1.174-10.960 | **0.025** |
| **HVPG, per mmHg** | 1.120 | 1.045-1.200 | **0.001** |
| **MELD, per point** | 1.125 | 0.967-1.310 | 0.128 |
| **Albumin, per g/dL** | 0.943 | 0.860-1.034 | 0.210 |
| **rs56163822 SNP (G/T vs. wild-type)** | 1.670 | 0.498-5.597 | 0.406 |
| **rs35724 SNP (G/C or C/C vs. wild-type)** | 0.411 | 0.191-0.885 | **0.023** |

Abbreviations: SNP – single nucleotide polymorphism; CPS – Child Pugh stage; HR – hazard ratio; 95%CI – 95% confidence interval; HVPG – hepatic venous pressure gradient; MELD – model of end stage liver disease

**Supplementary Table-4.** Distribution of *rs35724* variants among patients stratified by HVPG levels.

|  | **rs35724**  **wildtype**  **(G/G)** | **rs35724 polymorphism**  **(G/C, C/C)** | *P value* |
| --- | --- | --- | --- |
| ***HVPG 6-9mmHg, n=89*** | 36 (40.4%) | 53 (59.6%) | *0.175* |
| ***HVPG 10-20mmHg, n=215*** | 64 (29.8%) | 151 (70.2%) |  |
| ***HVPG >20mmHg, n=98*** | 35 (35.7%) | 63 (64.3%) |  |
| ***No CSPH, n=89*** | 36 (40.4%) | 53 (59.6%) | *0.120* |
| ***CSPH (HVPG ≥10mmHg), n=313*** | 99 (31.6%) | 214 (68.4%) |  |
| ***HVPG <16mmHg, n=204*** | 67 (32.8%) | 137 (67.2%) | *0.750* |
| ***HVPG ≥16mmHg, n=198*** | 68 (34.3%) | 130 (65.7%) |  |

Abbreviations: HVPG – hepatic venous pressure gradient; CSPH – clinically significant portal hypertension (HVPG≥10mmHg)

**Supplementary Figure-1.** Kaplan-Meier analyses on any (further) hepatic decompensation **A** in patients with and without *rs56163822* SNP in the overall cohort and **B** in patients with CPS-A.


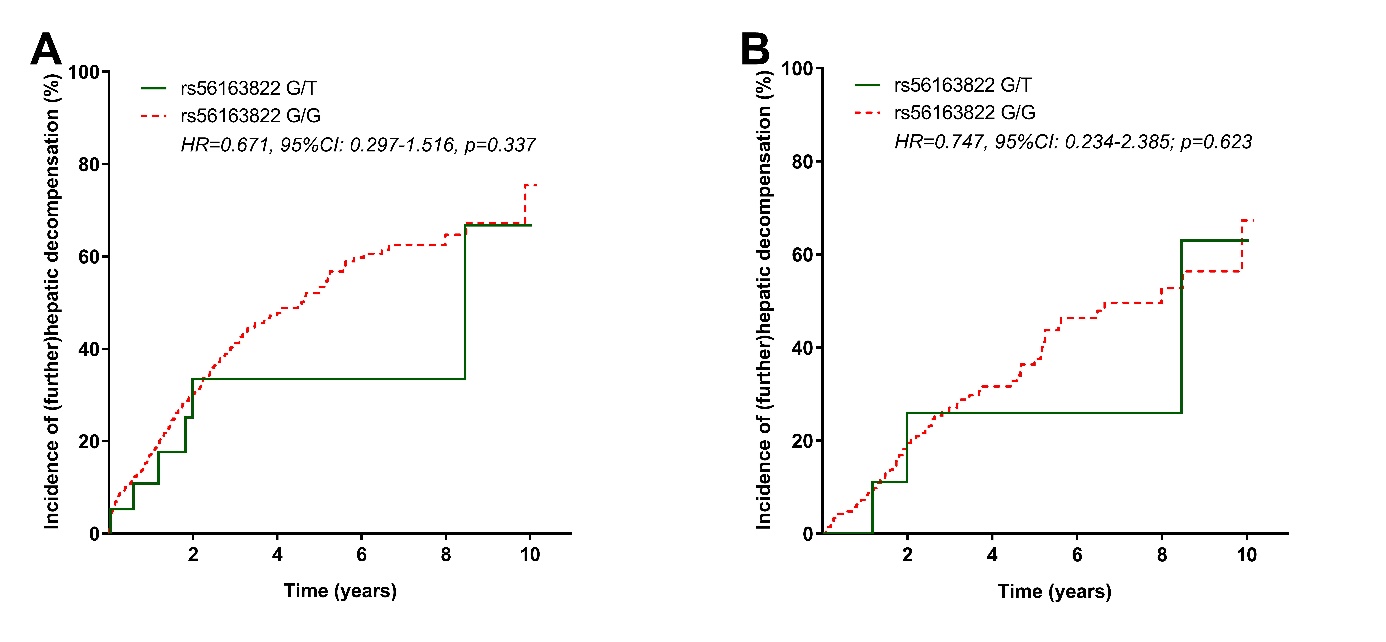


Abbreviations: SNP – single nucleotide polymorphism; CPS – Child Pugh stage; HR – Hazard Ratio, 95%CI – 95% confidence interval

**Supplementary Figure-2.** Kaplan-Meier analyses on the incidence of **A** large-volume paracentesis, **B** hepatic encephalopathy, **C** spontaneous bacterial peritonitis and **D** portal hypertensive bleeding in patients with CPS-A and *rs35724* minor allele.


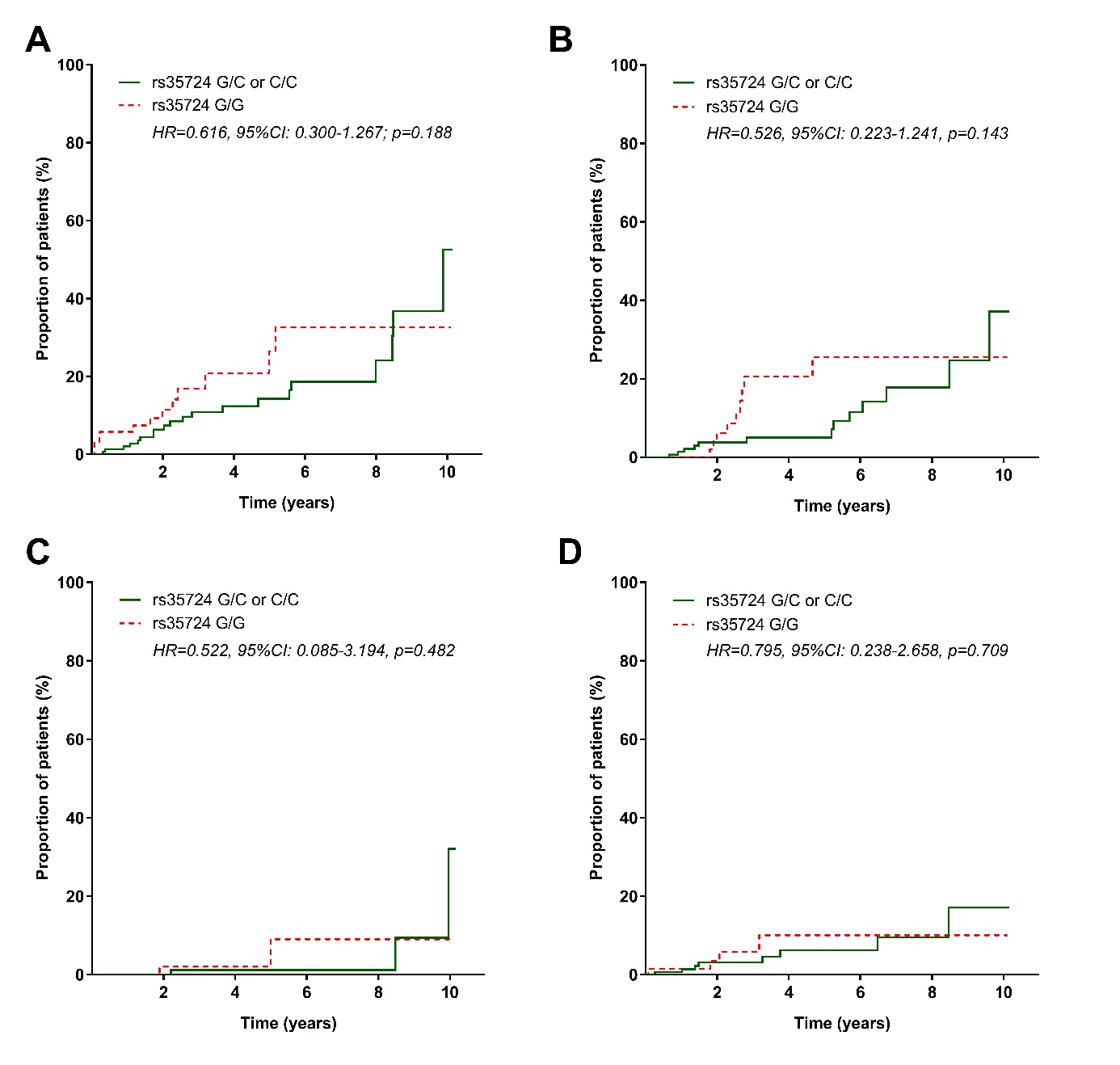


Abbreviations: SNP – single nucleotide polymorphism; CPS – Child Pugh stage; HR – Hazard Ratio, 95%CI – 95% confidence interval

**Supplementary Figure-3.** Transplant-free survival* in patients with and without *rs56163822* SNP **A** in the overall cohort and **B** in patients with CPS-A.


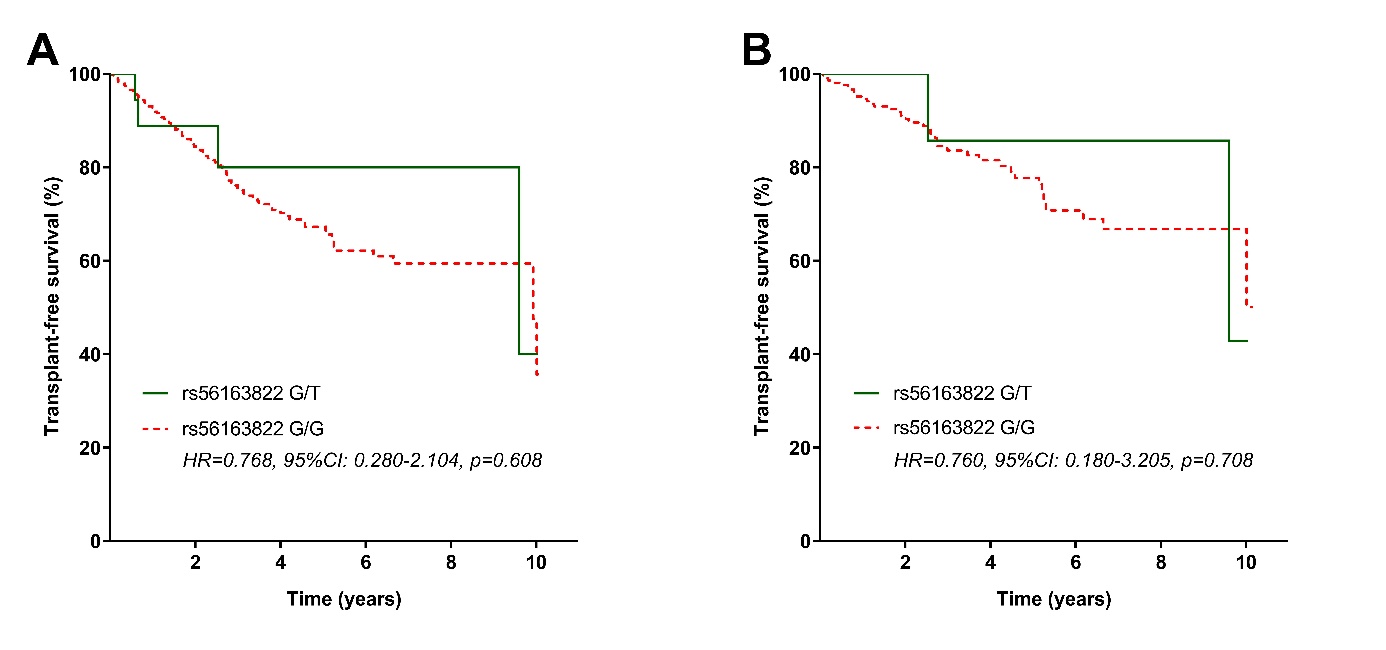


*: Patients were censored at the day of liver transplantation, non-liver-related death, or end of follow-up

Abbreviations: SNP – single nucleotide polymorphism; CPS – Child Pugh stage; HR – Hazard Ratio, 95%CI – 95% confidence interval

**Supplementary Figure-4.** Kaplan Meier analyses on transplant-free survival **A** in female patients and **B** in female CPS-A patients with *rs35724* SNP minor allele.


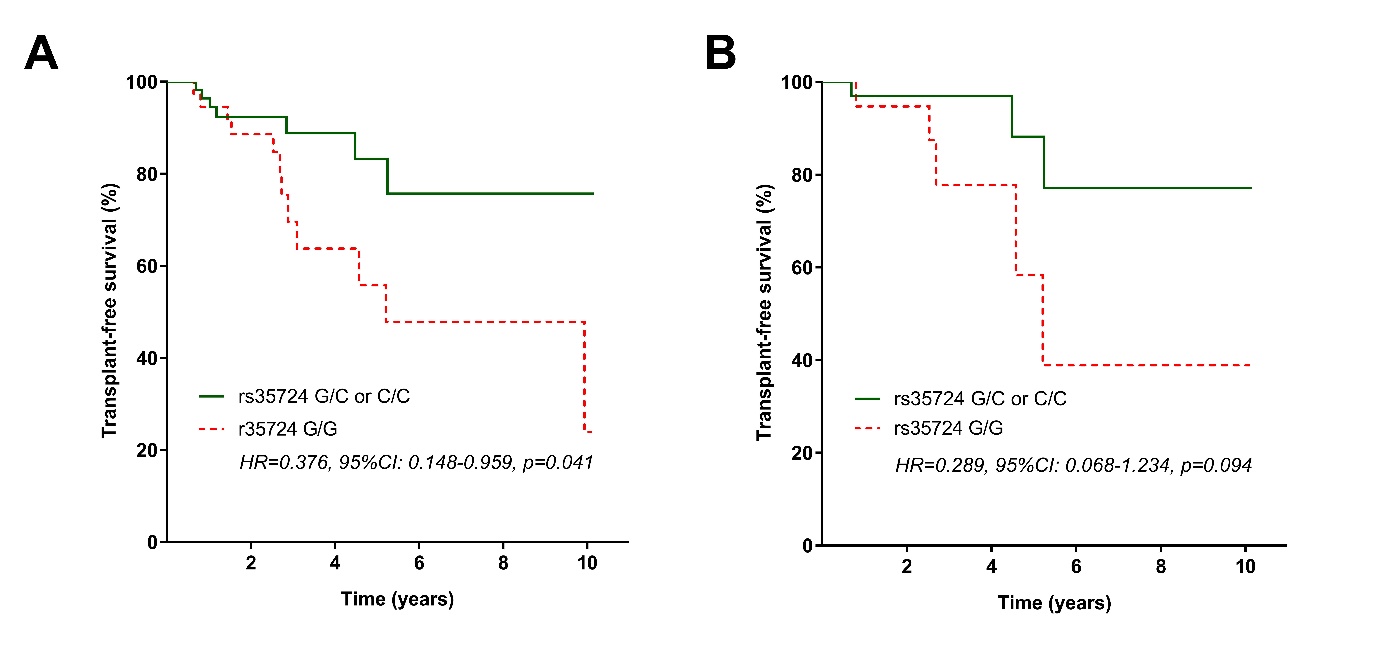


Abbreviations: SNP – single nucleotide polymorphism; CPS – Child Pugh stage; HR – Hazard Ratio, 95%CI – 95% confidence interval

**Supplementary Figure-5.** Kaplan-Meier analyses on (further) hepatic decompensation **A** in patients with mild portal hypertension (HVPG 6-9mmHg), **B** in patients with HVPG 10-20mmHg, **C** in patients with high risk CSPH (HVPG >20mmHg) as well as **D** in patients with clinically significant portal hypertension (CPSH, HVPG ≥10mmHg) and **E** HVPG ≥16mmHg, stratified according to presence of *rs35724* minor allele.


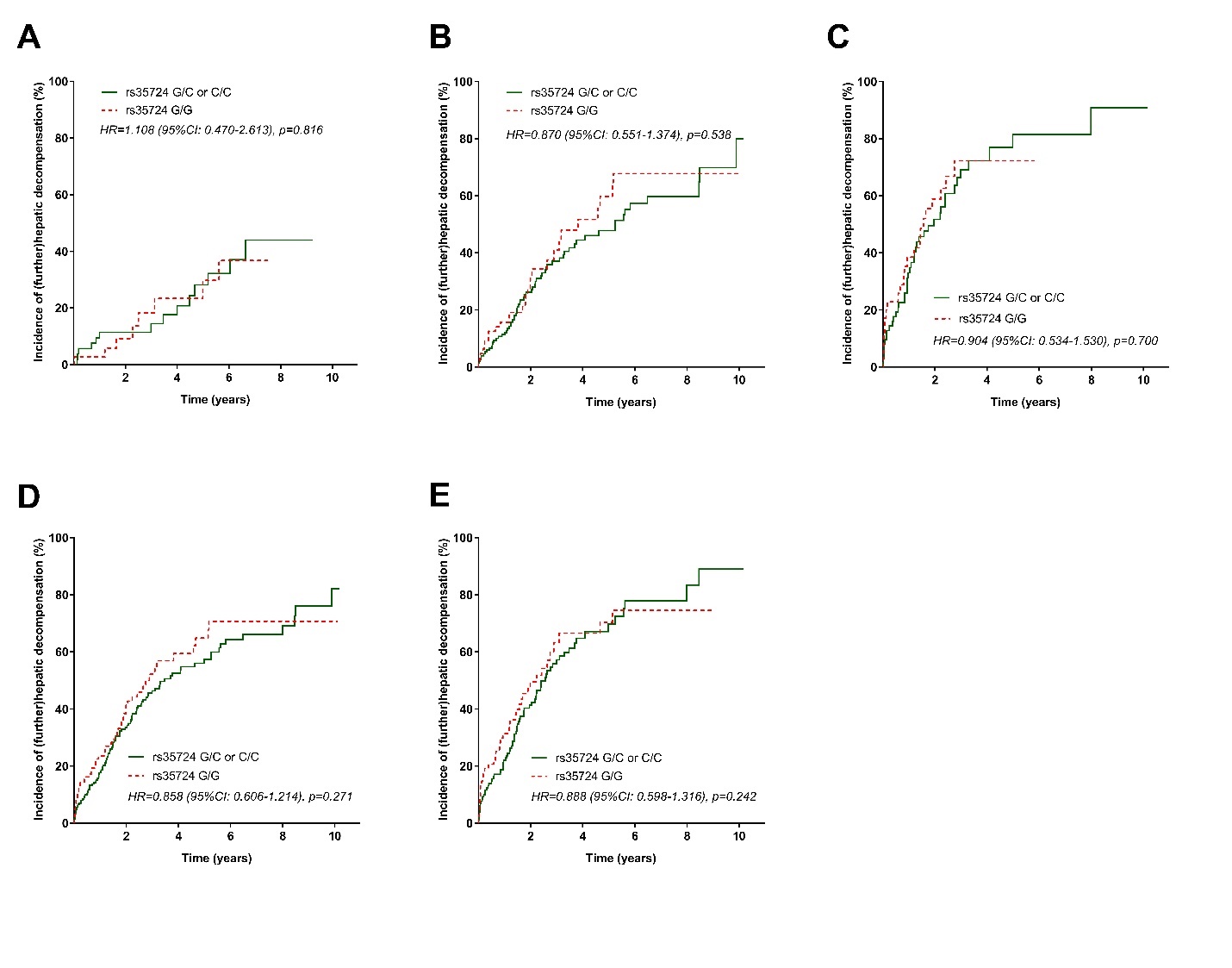
*: Patients were censored at the day of liver transplantation, non-liver-related death, or end of follow-up

Abbreviations: HVPG – hepatic venous pressure gradient; CSPH – clinically significant portal hypertension; HR – Hazard Ratio, 95%CI – 95% confidence interval
